# Effects of Chain Length of Chitosan Oligosaccharides on Solution Properties and Complexation with siRNA

**DOI:** 10.3390/polym11081236

**Published:** 2019-07-25

**Authors:** Tim Delas, Maxime Mock-Joubert, Jimmy Faivre, Mirjam Hofmaier, Olivier Sandre, François Dole, Jean Paul Chapel, Agnès Crépet, Stéphane Trombotto, Thierry Delair, Christophe Schatz

**Affiliations:** 1 Laboratoire de Chimie des Polymères Organiques (LCPO), Univ. Bordeaux, CNRS, Bordeaux INP, UMR 5629, 33600 Pessac, France; 2 Ingénierie des Matériaux Polymères (IMP), CNRS UMR 5223, Université Claude Bernard Lyon 1, 69622 Villeurbanne, France; 3 Centre de Recherche Paul Pascal (CRPP), UMR CNRS 5031, Univ. Bordeaux, 33600 Pessac, France

**Keywords:** chitosan, siRNA, oligosaccharide, polyelectrolyte, complexation, electrostatics

## Abstract

In the context of gene delivery, chitosan has been widely used as a safe and effective polycation to complex DNA, RNA and more recently, siRNA. However, much less attention has been paid to chitosan oligosaccharides (COS) despite their biological properties. This study proposed to carry out a physicochemical study of COS varying in degree of polymerization (DP) from 5 to 50, both from the point of view of the solution properties and the complexing behavior with siRNA. The main parameters studied as a function of DP were the apparent pK_a_, the solubility versus pH, the binding affinity with siRNA and the colloidal properties of complexes. Some parameters, like the pK_a_ or the binding enthalpy with siRNA, showed a marked transition from DP 5 to DP 13, suggesting that electrostatic properties of COS vary considerably in this range of DP. The colloidal properties of siRNA/COS complexes were affected in a different way by the COS chain length. In particular, COS of relatively high DP (≥50) were required to form small complex particles with good stability.

## 1. Introduction

Chitosan, the chitin partially deacetylated derivative composed of β-(1→4)-linked d-glucosamine (GlcN) and *N*-acetyl-d-glucosamine (GlcNAc) groups, is a biopolymer of major importance with applications spanning water treatment, agriculture, cosmetics, the food industry and biopharmaceutics [1]. Among these, chitosan is well-known for its potential in biomedical applications owing to its biocompatibility, biodegradability and bioactivity including bacteriostatic, healing, immunologic and antitumoral activities [2]. In particular, chitosan has been used as a polycation for the design of non-viral systems for DNA delivery and more recently, for small-interfering RNA (siRNA) [3,4,5,6,7,8]. These systems rely on the electrostatic complexation of the phosphate groups of nucleic acids with the protonated amine functions of chitosan under mild acid conditions. Unlike most polyamine systems used in gene delivery like polyethyleneimine or polylysine, chitosan has a low toxicity in relation to its low cationicity at physiological pH [9]. Several parameters related to the formation of chitosan/DNA (siRNA) polyplexes have been studied with the aim of increasing the stability of complexes toward dissociation, the resistance to serum proteases, the escape from the immune system, cell targeting, cell internalization and the endosome escape. The main parameters include: The degree of protonation of chitosan related to the pH of the medium; the amine to phosphate molar ratio (N:P); the degree of *N*-acetylation of chitosan (DA); and the molar mass of chitosan [6,7,10,11,12]. The precise control of these four parameters enables: The size and surface charge of polyplexes to be adjusted; the binding affinity with nucleic acids; and the resulting stability of complexes. In the case of siRNA delivery, the size of polyplexes is primarly determined by the chitosan chain length;low molar mass chitosan usually give rise to small-sized complexes [13,14]. However, different studies have demonstrated that chitosans of low molar mass (<10 kDa) have difficulty in forming stable complexes, especially in the presence of serum, where destabilization through competitive plasma protein displacement may occur [11,13,14]. For chitosans of higher molar mass (>10 kDa), the chain length has little effect on the polyplexes stability, the cell uptake and the gene knockdown [14]. The latter is primarily determined by the surface charge of the polyplexes which is related to the degree of acetylation and pH. A knockdown efficiency higher than 70% was reported with an almost fully *N*-deacetylated chitosan (DA of 2%) [14]. However, no significant effect of the (N:P) ratio above 5 was evidenced on the size, colloidal stability and gene knockdown [14].

This work investigated the role of the chitosan chain length on the complexing properties with siRNA and attempts were made to correlate it with the solution behaviour of chitosan chains alone. For this purpose, this study used chitosan oligosaccharides (COS) of low DA (<1%) with degrees of polymerization (DPs) comprised between 5 and 50. Even though low molar mass chitosans are not best suited for siRNA delivery, it is important to understand, at least from a fundamental point of view, the role of the DP on the affinity with siRNA and the resulting stability of complexes. In this study, the physicochemical properties of COS were studied by potentiometric titration and dynamic light scattering (DLS). Then, the properties of COS-siRNA complexes were assessed by DLS, siRNA assay and isothermal titration calorimetry. The COS were always used in their fully protonated state to study complexation with siRNA under the best conditions. An important aspect of this work is to determine whether an oligomer-to-polymer transition can be evidenced in the cooperative binding of chitosan with siRNA, as already demonstrated with DNA [15].

## 2. Materials and Methods

**siRNA.** The siRNA duplex was provided by Kaneka Eurogentec S.A. (Seraing, Belgium) and received as lyophilisate after purification by reverse phase HPLC. The sense sequence was 5′-[Phos]GUCUCAGCCUCUUCUCAUUCCUG[dC][dT]-3′ (M = 7087.6 g/mol) and the antisense was 5′-AGCAGGAA[mU]G[mA]G[mA]A[mG]A[mG]G[mC]U[mG]A[mG]A[mC][mA][mU][dT][dT] (M = 8999.6 g/mol). The molar masses were determined by MALDI-TOF analysis. The annealing step was performed by Eurogentec according to standard procedures.

**Chitosan oligosaccharides (COS).***N*-deacetylated chitosan (DA < 1%) with *M*_n_ ~ 90,000 g/mol and Đ = 2.1 (Figure S1) was provided from Mahtani Chitosan (batch 20140503). A nitrous acid depolymerization was performed according to the method proposed by Allan and Peyron [16,17]. Chitosan was dissolved at 3 wt % in water in the presence of a stoichiometric amount of HCl relative to amine functions. After 3 days of vigorous stirring, sodium nitrite was added to the chitosan solution at various nitrite/GlcN molar ratios depending on the targeted DP. The reaction proceeded under magnetic stirring in a closed reaction vessel for 24 h at room temperature. After filtration on a 1.0 µm pore size glass fiber membrane (Pall), the solution was lyophilized and the lyophilisate was dissolved in a minimum volume of ultrapure water. The COS were precipitated in ethanol:acetone (1:1 *v*:*v*) mixture and the precipitate was washed three times with the same solvent mixture and centrifuged. The swollen oligomers were then repeatedly (3×) dissolved in ultrapure water and lyophilized. The final mass yield of purified COS under their hydrochloride salt form was above 70%.

**COS characterization by ^1^H NMR and SEC-MALLS.** The chemical structure of COS was determined at 298 K from proton nuclear magnetic resonance (^1^H-NMR) spectroscopy on a Bruker AVANCE III HD 400 MHz spectrometer using a 5 mm Bruker multinuclear z-gradient direct probe. The ^1^H NMR spectra were calibrated from the signal of HOD at 4.79 ppm [18]. The degree of polymerization (DP) of COS which corresponds to the number of glucosamine (GlcN) units within the COS chain was obtained from the integral ratio of the H-2 protons of GlcN units at 3.20 ppm and the H-1 or H-3 protons of the terminal 2,5-anhydro-D-mannose (M-unit) located at 5.11 and 4.46 ppm, respectively [19]. Note that in the ^1^H-NMR analysis conditions used here, the aldehyde group of the M-units does not exist in its free (–CHO) form but exclusively in its hydrated (–CH(OH)_2_) form [20]. The mass-average molar mass (*M*_w_), the number-average molar mass (*M*_n_) and the dispersity (Đ) of COS were determined by high-performance size exclusion chromatography (SEC) (UltiMate 3000 HPLC, Thermofisher, Waltham, MA, USA) with a multiangle laser light scattering detection (MALLS) (Dawn Heleos, Wyatt, Santa Barbara, CA, USA) operating at λ_0_ = 658 nm and a differential refractive index detector (Optilab rEX, Wyatt) operating at the same wavelength. The COS samples were separated on two serially connected columns (Tosoh TSK gel columns, G4000PWXL & G3000PWXL, Tokyo, Japan). A degassed 0.3 M acetic acid/0.2 M sodium acetate buffer (pH 4.5) was used as eluent after filtration on a 0.22 µm pore size membrane (Millipore). The flow rate was maintained at 0.6 mL/min, and the amount of sample injected was 100 µL at a concentration of 5 mg/mL. The values of the refractive index increment (dn/dc) were independently determined from a range of five chitosan concentrations for each DP with the Optilab reX refractive index detector using same conditions for SEC-MALLS analysis but without degassing the buffer. The values of the number-average degree of polymerization (DP_n_) of COS were derived from SEC-MALLS analysis by using the following relation: DP_n_ = (*M*_n_ − *M*_M-unit_)/*M*_GlcN_ where *M*_M-unit_ is the molar mass of the terminal 2,5-anhydro-D-mannose unit and *M*_GlcN_ is the molar mass of the glucosamine unit.

**Thermogravimetric Analysis.** The thermogravimetric analyses were performed using a Q50 device (TA instruments, New Castle, DE, USA) in order to determine the water content of chitosan samples. It was operated from 25 to 150 °C at a ramp temperature of 2 °C/min under a flow of nitrogen. The values of water content were taken into account in the preparation of the COS solution.

**Potentiometric Titration.** The potentiometric titrations of COS varying in DP were performed using an automatic pH titrator (TitroLine 7800, SI Analytics, Mainz, Germany) equipped with a microelectrode. The COS solutions were prepared at 1 g/L in water and the pH was adjusted to 3.0 with a known amount of HCl 0.1 M to ensure the full protonation of glucosamine residues. The titrations were performed with 10 mL of COS solution using 0.1 M NaOH (NIST Standard Concentrate, for Volumetric Analysis, Fisher Chemical, Pittsburgh, PA, USA). The volume of each injection of NaOH was set to 10 µL at a rate of 5 mL/min and the time between each injection was set to 60 s. The apparent values of pK_a_ were derived from the Henderson-Hasselbalch equation:

(1) $$pK_{a}=pH+log\frac{1-\alpha }{\alpha }$$

where *α* is the degree of dissociation of the ammonium groups defined as:

(2) $$\alpha =\frac{\left[ChitNH_{2}\right]}{\left[ChitNH_{3}^{+}\right]+\left[ChitNH_{2}\right]}$$

and according to the requirement of the electroneutrality:

(3) $$\left[ChitNH_{3}^{+}\right]=\left[Cl^{-}\right]+\left[OH^{-}\right]-\left[Na^{+}\right]-\left[H_{3}O^{+}\right]$$

Note that Cl^−^ ions come from both the added HCl and the glucosamine hydrochloride residues. All concentrations were corrected by the dilution factor related to the titration. In addition to the degree of dissociation, the values of the degree of protonation (γ) of the COS were also determined from the titration data:

(4) $$\gamma =\frac{\left[ChitNH_{3}^{+}\right]}{\left[ChitNH_{3}^{+}\right]+\left[ChitNH_{2}\right]}$$

**Solubility of COS as function of pH**. The COS solutions were prepared at 1 g/L in deionized water and filtered on 0.22 µm cellulose acetate filter. The pH of the COS solutions increased by adding 0.1 M NaOH, and aliquots were taken at various pHs. The scattered intensity (kilo counts per second, Kcps) of the COS solutions was determined at 25 °C using a ZetaSizer Nano ZS (Malvern Panalytical Ltd., Malvern, UK) working at λ = 632.8 nm with a back scattering angle detection (173°).

**Preparation of COS/siRNA complexes**. The complexes between siRNA and chitosan were formed by mixing 50 µL of COS solution with 50 µL of siRNA solution, both prepared in RNase free water. The concentration of the siRNA solution was set at 0.1 g/L (0.294 mM in phosphate groups), while the COS concentration in protonated glucosamine residues was varied according to the targeted nitrogen to phosphate (N:P) ratio. The COS solution was filtered on 0.22 µm cellulose acetate membrane prior to use. The complexation was carried out by a one shot addition with a micropipette of the COS solution to the siRNA solution for N:P < 1. The opposite order of addition was used for N:P > 1. By doing sothe component in default was always added to the one in excess. Hencethe system avoided the experience of the neutral state at charge stoichiometry [21,22]. The fast mixing conditions were obtained by achieving the complexation reaction in a 1.5 mL plastic microtube placed in a VXR basic Vibrax (IKA) set at 1000 rpm. The particles of complexes were analyzed by dynamic light scattering using a ZetaSizer Nano ZS (see above) at 25 °C. Further, three to five measurements were performed for each condition of complexation. The particle size distribution was determined using a non-negative least squares (NNLS) algorithm with a quadratic weighting scheme, which places more emphasis on fitting the earlier channels where the signal intensity is the greatest.

**siRNA assay with toluidine blue (TB)**. The amount of complexed siRNA was assayed with toluidine blue O (Sigma Aldrich) according to a depletion protocol. The dispersions of complexes prepared at various N:P ratios were incubated (30 min) in 1.4 mL of 0.1 M acetate buffer pH 4.0 containing an excess of TB. After centrifugation (20 min at 14000 rpm), free TB was titrated in 200 µL of supernatant by absorbance measurements at λ = 585 nm using a microplate reader (SpectraMax M2, Molecular Devices, San Jose, CA, USA). The free siRNA concentration in the complexes was determined via calibration curves established in similar conditions (Figure S8). The amount of complexed siRNA was then deduced by a simple difference between the initial and final siRNA concentrations in the complex dispersions. All experiment were performed in triplicate.

**Isothermal Titration Calorimetry.** The binding studies were performed using a Microcal iTC200 (Malvern Panalytical Ltd., Malvern, UK). All titrations were performed at 25 °C in 10 mM acetate buffer at pH = 4.5. Further, 16 consecutive injections of 2 µL (0.4 µL for the first injection) of COS solution at a concentration of 10 mM in GlcN units were performed in 200 µL of siRNA solution at a concentration of 0.7 mM in the phosphate groups. The injections were made at intervals of 180 s. All solutions were degassed before use. The COS solutions were also injected into the buffer as blank titrations. All experiments were carried out in duplicate. The thermograms were analyzed using a modified multiple non-interacting sites (MNIS) model (see details in Supporting Information) [23].

## 3. Results

### 3.1. Solution Properties of Chitosan Oligosaccharides

**Preparation of a library of COS.** Chitosan oligosaccharides were prepared by depolymerization of an almost fully *N*-deacetylated chitosan (DA < 1%) of 9 × 10^4^ g/mol with nitrous acid (Figure S1). In this reaction, the number of broken glycosidic linkages is approximately stoichiometric to the amount of nitrous acid used. After several purification steps, the COS were obtained under the water-soluble hydrochloride salt form. All chemical shifts could be assigned on ^1^H NMR spectra performed in D_2_O (Figure S2). In particular, the signal at 5.11 ppm was assigned to the gem diol group of the terminal 2,5-anhydro-D-mannose (M-unit) [19]. Importantly, no trace of hydroxymethylfurfural (HMF), the main degradation product of the M-unit, was evidenced by the absence of the characteristic peaks of HMF at 9.52, 7.52, 6.66 and 4.68 ppm [19]. The COS were used without reducing the M-unit with sodium borohydride (NaBH_4_) or sodium cyanoborohydride (NaBH_3_CN) as it has been recently shown that the reduction step could significantly increase the COS molar mass through side-branching reactions involving the aldehyde group of the M-unit and amino groups of the COS chain [24]. The average values of the molar mass, the degree of polymerization and dispersity obtained through SEC-MALLS and ^1^H NMR are given in Table 1. The mass recoveries derived from the mass injected and the dn/dc values of COS were always higher than 80%, thus emphasizing the absence of strong enthalpic interactions between the macromolecules and the column packing. Similar values of DP were obtained by SEC and NMR, with the exception of the largest COS where NMR provided a higher DP value. This may be due to the uncertainties associated with the baseline determination and integral limits by NMR, particularly when the signal of the terminal M-unit is of low intensity.

**Figure 1:**
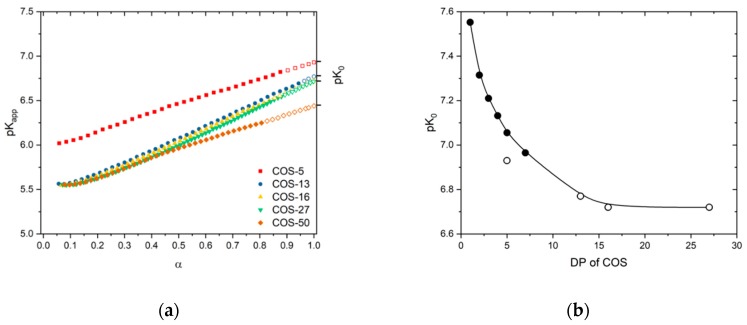
The variation of the apparent pK_a_ of COS as a function of the degree of dissociation (**a**). The open symbols correspond to extrapolated values. The concentration of COS is 1 g/L. (**b**) The variation of the intrinsic dissociation constant (pK_0_) as a function of the degree of polymerization (DP) (open symbols). The data are plotted together with those in Ref. [26] obtained on isomolecular COS fractions of DP 1 to DP 7 (closed symbols). The line was added to guide the eye.

**Potentiometric titration of COS**. The pK_a_ values of COS were determined as a function of the dissociation degree of protonated glucosamine units (α) (Figure 1a) by applying the Henderson Hasselbalch equation (Equation (1)). For polyelectrolytes, only apparent pK_a_ values are obtained as the dissociation is influenced by the presence of existing charges on the chain. In the case of a polybase like chitosan, the expression of the apparent pK_a_ is [25]:

(5) $$pK_{app}=pK_{0}-\frac{0.4343}{RT}\left(\frac{\delta G_{el}}{\delta \alpha }\right)$$

where pK_0_, the intrinsic dissociation constant of an isolated protonated glucosamine group, is determined by extrapolating $pK_{app}$ to α = 1, and $\frac{\delta G_{el}}{\delta \alpha }$ is the electrostatic Gibbs free energy per unit of *α*. The latter term is related to the surface potential of the polyion, $\psi_{0}$, through $\frac{\delta G_{el}}{\delta \alpha }=eN_{a}\psi_{0}\left(\alpha \right)$ where e is the elemental electric charge and $N_{a}$ the Avogadro’s number.

By combining these two equations:

(6) $$pK_{app}=pK_{0}-\frac{0.4343}{kT}e\psi_{0}\left(\alpha \right)$$

Four main results can be drawn from the Figure 1a. First, the increase of pK_app_ with α emphasizes a polyelectrolyte behavior in contrast to d-glucosamine characterized by a single pK_a_ value of 7.55 [26]. The origin of this behavior originates from the electrostatic repulsion among neighboring charges which makes the energy cost of protonation higher at low degrees of dissociation. A similar trend has been reported for chitosan [9,27,28,29], chitosan oligomers [26,30] and other polyamines [31]. Second, the pK_app_(α) variations are linear which suggests that COS chains do not undergo significant conformational change during titration. However, it was observed that the pK_app_ variation tends to flatten at high values of α due to the polymer precipitation. In such cases, the values of pK_app_ were extrapolated to α =1 from the linear part of the plots. Third, the decrease in pK_app_ values with the DP, already observed for chitosan oligomers [26,32], means that increasing the COS chain length has same effect on the electrostatic potential of the COS chains as decreasing α, that is, favoring the proton dissociation by electrostatic repulsion between charged units. Finally, COS-5 has distinct behavior from COS-13-16-27-50 as shown by the much higher pK_app_ values across the range of α, suggesting that a marked transition occurred between these two regimes of DP in relation with the increase of the electrostatic potential of the chains.

Regarding the pK_0_ values, COS-5 had a pK_0_ of 6.93, while pK_0_ values of 6.77 to 6.72 were found for COS-13-16-27 (Table 2). For COS-50, the pK_0_ of 6.44 was probably underestimated because the pK_app_ variation strongly deviated from the linearity around α = 0.5 (i.e pH = 6.0) due to decreased solubility of COS [29]. The pK_0_ values were plotted together with those found by Tsukada and Inoue on pure COS fractions ranging from DP 1 to DP 7 (Figure 1b) [26]. The authors have interpreted the progressive decrease of pK_0_ with DP as a chain-end effect, since the intrinsic dissociation constant of the internal residues of the COS chain was lower than the terminal residues. The values of 6.74 and 7.62 were respectively derived for the internal and terminal residues [26]. The value of 6.74 agrees well with the pK_0_ found for COS-16 and COS-27 where chain end effects can be neglected. From Figure 1b, it appears that increasing the chain length above DP 10 does not significantly modify the values of pK_0_.

The titration data were also fitted with the extended Henderson-Hasselbalch equation introduced by Katchalsky and Spitnik [33]:

pH = pK_1/2_ + n log [α/(1 − α)] (7)

where pK_1/2_ is the pK_a_ at α = 0.5 and n an empirical parameter related to intramolecular interaction in chitosan chains [34]. For monomeric acids or bases, n is equal to 1. The values of n higher than 1 reflect electrostatic interactions between neighboring charged groups. For the COS-5, n = 1.53, while n~1.73 for the COS-13-16-27 (Table 2, Figure S3) which is again consistent with the existence of two distinct regimes of DP. A similar trend was observed for the variation of pK_1/2_. From these results, it can be concluded that the gradual increase of the electrostatic free energy of COS with the chain length results in anti-cooperative proton binding to COS due to the increased repulsions between charged units. The range of the electrostatic interactions in solution can be evaluated though the Debye length ($\lambda_{D}$) defined by:

(8) $$\lambda_{D}={\left(8{\pi l}_{B}I\right)}^{-1/2}$$

with $l_{B}$, the Bjerrum length and I the ionic strength $\left(I=\frac{1}{2}\underset{i}{\sum }c_{i}z_{i}^{2}\right)$. In the conditions of concentration used for the titration of COS, $\lambda_{D}$ = 3.5 nm which corresponds to a segment of 7 units of COS regularly aligned, considering a length per unit of chitosan of 0.51 nm [35]. As the decrease in pK_a_ was most significant between DP1 and DP7 (Figure 1), the proton binding to COS chains must be mainly governed by the extent of electrostatic interaction evaluated by $\lambda_{D}$. In the presence of added salt, the Debye length is decreased which results in a dramatic increase of the apparent pK_a_ in the whole range of α values [9].

**Figure 2:**
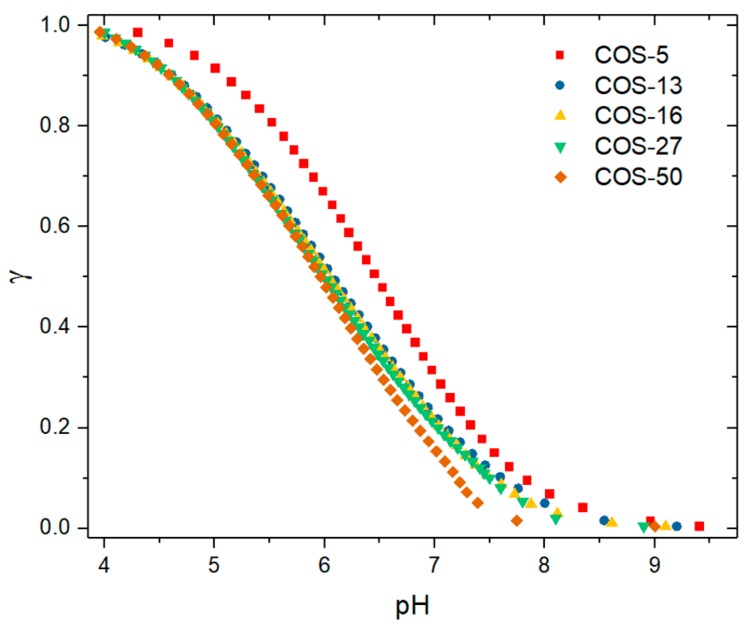
The degree of protonation (γ) as function of the pH for COS varying in DP. The concentration of COS is 1 g/L.

Eventually, the degree of protonation (γ) of COS as a function of pH was also derived from potentiometry data (Figure 2). All COS samples were fully protonated at pH 4, while less than 20% of the glucosamine units were still charged at pH 7.4, suggesting low solubility of COS under such conditions (see below).

**Figure 3:**
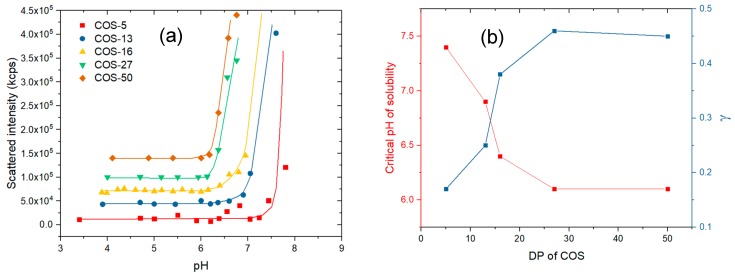
(**a**) The variation of the scattered light intensity (in kcps, kilo count-per-second) of COS solutions at 1 g/L as a function of the pH. The plots are shifted along the y-axis for better visibility; (**b**) the variation of the pH at the onset of the COS precipitation as a function of the DP. The corresponding values of degrees of protonation (γ) at the critical pH are also plotted (see Figure 2). The lines were added to guide the eye.

**Solubility behavior of COS.** The hydrochloride form of COS is completely soluble in water, giving a pH of 4 for solutions prepared in dilute conditions. The solubility of COS as a function of pH was determined by light scattering (Figure 3a). Light scattering is a more sensitive technique than transmittance to detect aggregation and precipitation phenomena owing to the sixth power dependence of the particle radius to the scattered intensity (Rayleigh scattering). Three main results can be drawn from the Figure 3: (i) The onset of chitosan precipitation is observed within a narrow range of pH values in agreement with previous studies (Figure 3a) [36,37]; (ii) the corresponding critical pH values are lowered when the DP is increased and becomes constant from DP 27 (Figure 3b); (iii) the degree of protonation (γ) at the critical pH follows the opposite trend, namely γ increases with the DP and becomes constant from DP 27. The differences observed in the pH solubility profiles are in agreement with the respective pK_a_ values of COS, that is, the higher the pK_a_, the higher the solubility. The increase with DP of the degree of protonation at the critical pH may emphasize a stronger involvement of cooperative hydrogen bonding in the precipitation of chitosan, associated with a less unfavorable mixing entropy.

### 3.2. Complexation of COS with siRNA

The complexation of COS under their hydrochloride form with siRNA was perfomed at various N:P ratios in water without added salt in order to maximize the formation of salt bonds between the two polyions. The complexes obtained from siRNA and chitosan can be classified as strong in the sense of the ion pairing between opposite charges is strong enough to hydrophobize the complexed polymer segments and promote their segregation into dense particles. As such, the complexes differ from coacervate systems where the ion pairing is less strong, resulting in more liquid-like structures [38]. The strong character of the complexation was well evidenced by the formation of a solid precipitate close to charge neutrality, i.e., at N:P = 1 (result not shown). In the presence of an excess of chitosan (N:P > 1) or siRNA (N:P < 1), the complexes can be stabilized by unpaired segments of excess polyion forming a stabilizing shell around the particles [22].

**Figure 4:**
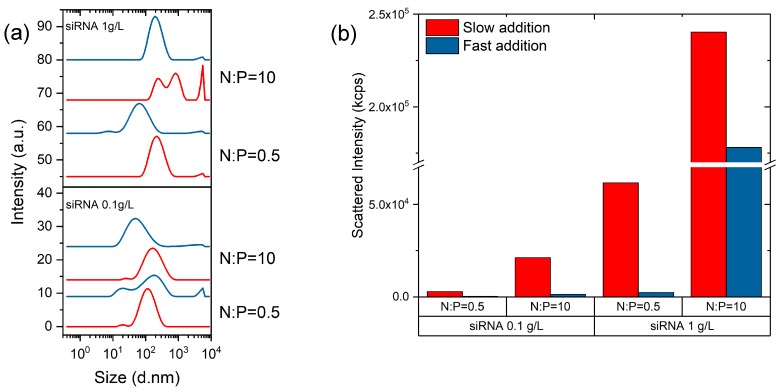
A dynamic light scattering (DLS) analysis of COS-50/siRNA complexes prepared by the dropwise addition (red) or one-shot injection (blue) of the component in default. The intensity-average size distributions of complexes (**a**) and the corresponding scattered intensities at 173° detection angle (**b**). The complexation was studied at two siRNA concentrations (0.1 g/L and 1 g/L) in RNase-free water and two N:P ratios (0.5 and 10).

It was noticed that the particle size distributions (PSDs) of complexes determined by DLS were dependent on the conditions of mixing of polyions, which suggests that complexes do not form equilibrium structures. As a consequence, much attention was paid to ensure the reproducible mixing conditions of COS and siRNA solutions. In addition, the COS solution was systematically added to the siRNA for N:P < 1, while the opposite mixing order was chosen for N:P > 1, so that the polyion in default was always added to that in excess. This mixing order prevents the system reaching the charge neutrality (N:P = 1) thus limiting aggregation [21,22]. The influence of the addition rate of the titrant on the scattered intensity and PSD of complexes was studied using the COS-50 at two N:P ratios (0.5 and 10) and two siRNA concentrations (0.1 and 1 g/L). The titrant was either added in a dropwise manner (slow addition) or by a one-shot injection with the micropipette (fast addition). As shown in Figure 4a, the fast addition always promoted the formation of complexes of smaller sizes and of lower scattered intensities compared to the slow addition. The polyelectrolyte complexation is a fast process with typical reaction times less than a millisecond [39,40]. Thus, in the case of a slow addition, the complexation medium was not homogeneized before the complexation started. The local over-concentrations of titrant could then result in the formation of larger and probably denser complexes, as shown by the higher scattered intensity values (Figure 4b). Conversely, a fast addition favoured the rapid homogeneization of the medium and the formation of less aggregated complexes. In this respect, the use of micromixers as those found in microfluidic chips should form complexes of the lowest achievable size [41,42,43].

**Figure 5:**
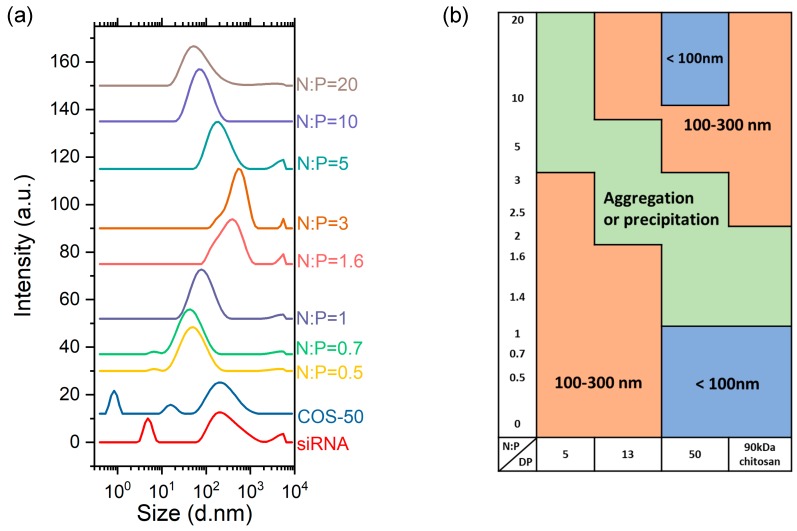
(**a**) The intensity-average particle size distributions of COS-50/siRNA complexes in RNase-free water by DLS with a 173° angle detection at various N:P ratios using a siRNA concentration of 0.1 g/L. (**b**) The particle size map representing the typical values of hydrodynamic diameter (D_H_) of complexes obtained at various N:P ratios with COS varying in DP and parent chitosan (90 kDa).

**Size distribution of complexes by dynamic light scattering**. The intensity-average particle size distributions (PSDs) of COS/siRNA complexes were determined for four COS and parent chitosan (90 × 10^3^ g/mol) at various N:P ratios, from 0.5 to 20 (Figure S4). The complexes were always prepared by a fast addition of the component in default. Typical examples of PSDs obtained with the COS-50/siRNA at various N:P ratios are represented in Figure 5a together with the COS-50 and siRNA alone. For the latter, a hydrodynamic diameter of 5 nm could be determined in agreement with a previous study reporting a value of 4.2 nm for a duplex oligonucleotide of 20 base pairs [44]. The aggregates could also be detected, but they represented a negligible amount as shown by the absence of the aggregation peak in the number-average size distribution (Figure S5). A similar aggregation phenomenon was observed in 10 mM MES buffer at pH 6.2 (result not shown). For the COS-50, the peak detected at 15 nm must correspond to the COS chains (Figure 5a). Another peak was detected at 0.8 nm which was really small regarding the size of a saccharidic unit (0.51 nm) [35]. This peak was probably related to a fast diffusion mode where the motion of the polyion was coupled with the dynamics of small and much faster counterions [45]. In the presence of increasing amounts of salt, the fast mode progressively disappeared (Figure S6). Notably, the fast mode was not observed with siRNA, probably because of traces of salt in the sample. Despite the filtration of COS solutions, the PSD of COS-50 also showed an aggregation peak that was not detected in the number-average size distribution (Figure S5). Chitosan is known to have a strong tendency towards self-aggregation but this behaviour has been mostly documented for chitosans of relatively high MW and/or DA [46,47]. However, a few studies have reported the formation of weak aggregates in aqueous solutions of COS of low DA [32]. Specifically, Blagodatskikh et al. showed that COS of low MW (<12 kDa) and low DA (<3%) formed aggregates of constant sizes below the pK_a_ of chitosan. The aggregates that can be vizualized by electron microscopy are weakly bound as evidenced by their disruption under moderate shear stress [48] or in the presence of added salt (Figure S6). Here, the long-range electrostatic interaction with oppositely charged siRNA polyions is also expected to result in the aggregate disruption.

The PSDs of the complexes obtained with the COS-50 at various N:P ratios can be classified into three broad categories depending on the typical particle size found (Figure 5a). For N:P ≤ 1, small particles of the complex below 100 nm in diameter were detected. For 1 < N:P ≤ 3, the formation of some precipitates were observed highlighting the instability of the system near the charge neutrality. For N:P > 3, stable complexes of relatively small sizes were obtained. The evolution of the PSD with the N:P ratio was in agreement with a stabilization mechanism where the excess polyion, respectively the siRNA at N:P ≤ 1 and the COS-50 at N:P > 3, was located at the particle surface thereby preventing the aggregation of particles [49,50]. In most studies, N:P ratios ranging from 4 to 30 were used regardless the molar mass and DA of chitosan, thus emphasizing the limited stabilizing capacity of chitosan [3,14,51,52]. The PSDs were determined in a similar manner for COS varying in DP and parent chitosan at various N:P ratios (Figure S4). The role of the COS chain length on the complex formation is twofold: (i) It impacts the strength of the ion pairing with siRNA through a cooperative effect; (ii) it determines the stability of the complexes through the formation of a stabilizing shell around particles when COS is in excess. A map illustrating the correlation between the COS chain length and the complex size is represented in Figure 5b. For N:P ≤ 1 (excess siRNA), the size of the complex particles varied between 100 and 300 nm with COS-5 and COS-13, while smaller sizes (<100 nm) were obtained with COS-50 and the parent chitosan of 90 kDa. This can be interpreted by considering the affinity of COS with siRNA increases with the DP of COS as expected for a cooperative binding. As a result, the complexes obtained with larger COS may have a denser structure giving smaller sizes than those obtained with shorter COS. For N:P > 2, some aggregation was observed with COS-5-13, whereas the complexes formed with COS-50 and the parent chitosan were more stable, probably due to the more efficient electrosteric stabilization of long COS in excess. The most interesting system was obtained with the COS-50 at N:P 10 or N:P 20 where complexes of approximately 80 and 65 nm in diameter could be formed, respectively. The longer chitosan of 90 kDa did not form particles below 100 nm even at high N:P, which suggests that a compromise must be found regarding the optimal chain length of chitosan to achieve the stabilization of the complexes.

**Efficiency of the complexation.** The binding affinity of polyion to siRNA was determined by a direct titration of free siRNA in the complexation medium at various N:P ratios. This approach has been preferred to indirect methods, like the dye exclusion assay with ethidium bromide (EtBr), since the exclusion mechanism of the intercalated dye from siRNA (or DNA) is not completely known. In particular, it cannot be assumed that the dye displacement is stoichiometric with respect to the added amine groups. Nowadays, the RiboGReen RNA quantification kit is very popular to directly quantify siRNA via a calibration procedure. However, it was shown that reliable quantification of siRNA with RiboGreen could only be achieved under specific conditions of pH and ionic strength, particularly in Tris-EDTA buffer 1x at pH 8.0 where the mean fluorescence varied almost linearly with the siRNA concentration between 10 and 1000 ng/mL (Figure S7). In RNase-free water, used here as complexation medium, a strong curvature of the mean fluorescence as function of the siRNA concentration was obtained (Figure S7). In the acetate buffer pH 4.0, which is a suitable medium for complexation because COS are fully protonated at this pH, fluorescence was hardly detectable (Figure S7). In such conditions, the toluidine blue (TB), a conventional indicator in colloidal titration [53], was investigated to directly quantify siRNA in various solvent conditions [54,55,56]. The TB is a positively charged dye that can interact with phosphate groups of siRNA through electrostatic interaction [57]. A depletion approach was used in which the dispersions of complexes were first incubated with an excess of TB. After centrifugation, unreacted TB was titrated by absorbance measurements in the supernatants. As shown in Figure S8, a linear calibration curve of siRNA could be obtained in 0.1 M acetate buffer pH 4.0.

**Figure 6:**
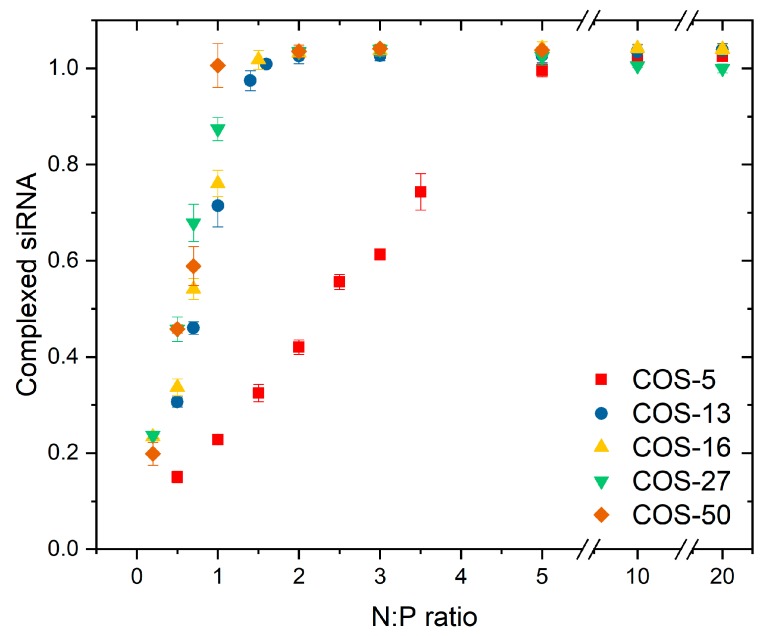
The fraction of complexed siRNA (ratio of the concentration of complexed siRNA to the total siRNA concentration) as function of the N:P ratio, determined by titration of free siRNA with toluidine blue in 0.1 M acetate buffer pH 4.0.

COS-13-16-27-50 have high affinity with siRNA, as evidenced by the Langmuir-like shape of the binding plots in Figure 6. The stoichiometry of complexation which refers to the number of formed salt bonds could be determined from the initial slope. Note that the stoichiometry of complexation differs from the composition of complexes where an excess of COS can be found for N:P > 1 (Figure 5). The initial slope is close to the unity for COS-13-16-27-50, indicating that the complexation stoichiometry is 1:1 (GlcN:phosphate) which is consistent with the full protonation of the COS at pH 4.0 (Figure 2). It confirms that the complexation is mainly of electrostatic origin, even though secondary interactions probably take place as well. Under such conditions, COS should be almost totally complexed for N:P < 1. No difference in affinity was detected with this method between COS-13-16-27-50. On the other hand, the binding of the COS-5 with siRNA is of much lower affinity as shown by the shape of the plot in Figure 6. A complexation stoichiometry of 5:1 (GlcN:phosphate) could be determined for this COS. These results are consistent with a cooperative binding mechanism of COS with siRNA, that is, the higher the DP the stronger the binding. A minimum of 13 units of COS is required here to achieve a stoichiometric complexation with siRNA. By using a larger library of COS samples, the critical DP could be determined more precisely, with the actual value probably between 5 and 13.

**Figure 7:**
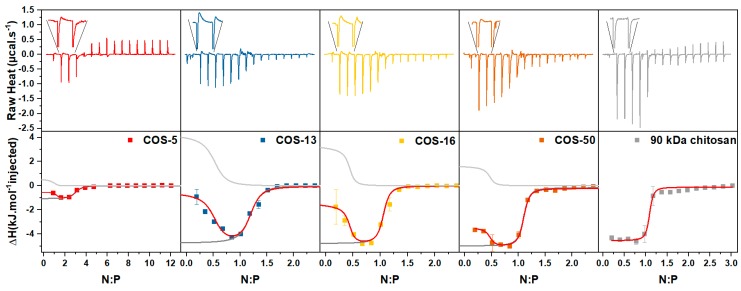
(Upper panel) The heat flow per injection versus time for the isothermal titration of siRNA (0.7 mM) by COS varying in DP (10 mM). The first injection peaks are magnified to illustrate the presence of an endothermic component superimposed on the main exothermic one. (Lower panel) The integrated heats of binding versus the N:P ratio. The continuous red lines represent the overall fit to the experimental data. The dark grey and light grey lines represent the ion-pairing and the aggregation contributions to the overall process, respectively. All titrations were performed at 25 °C in 10 mM acetate buffer at pH 4.5.

**Thermodynamics of complexation**. Isothermal titration calorimetry (ITC) was used to study the thermodynamics of complexation of siRNA with COS varying in DP. All experiments were performed in 10 mM acetate buffer at pH 4.5 which has a low ionization enthalpy [58] to prevent heat effects associated with proton exchange during the complexation. The experimental data represented in Figure 7 indicate that the complexation was always exothermic which is in agreement with the data of the literature [6,59]. However, non-sigmoidal shapes of titration curves were observed with the exception of chitosan of higher molar mass. Under such conditions, the data could not be properly fitted with a single set or two sets of identical sites model. However, a close inspection of the ITC thermograms of COS of low DP reveals that the initial peaks consisted of a narrow and negative component followed by another one, broad and positive (inserts in Figure 7). The first component (exothermic) was attributed to the electrostatic ion pairing of protonated glucosamine units with phosphate groups. The different possibilities were considered for the second one (endothermic). First, the blank titration, i.e., the injection of COS in the acetate buffer was endothermic because the hydrochloride form of the COS slightly deprotonated upon dilution in the buffer at pH 4.5 (Figure S9) [59]. However, this effect should be negligible as the heat of dilution was subtracted from the integrated peak area. In addition, the dilution peaks are rather narrow (Figure S9) compared to the broad endothermic peaks observed in Figure 7. Second, the pK_a_ of chitosan is expected to increase upon complexation with siRNA for the same reason that the pK_a_ increases with α (Figure 1a) [60]. However, this effect should give negative peaks as the protonation of chitosan is exothermic [59]. In addition, since COS were almost completely protonated in the acetate buffer, such effects must be negligible. Third, the broad shape of the endothermic peaks could be the signature of an aggregation process through hydrophobic interaction for which the unfavorable enthalpy arises from the energy associated with the disruption of structured water molecules. Due to its position away from stoichiometry, the aggregation peak differs from the coacervation or precipitation peak that can be observed for synthetic polyelectrolyte systems at neutrality [23,61,62]. It also differs from the so-called condensation peak (endothermic) attributed to the collapse of DNA chains after neutralization of phosphate groups by cationic ligands [63,64]. Here, the aggregation was stronger for smaller COS as seen by the more pronounced V shape on the thermograms of COS 13 and COS 16. It suggests that the complexes obtained from shorter COS were not stable at low N:P which is in agreement with previous observations by DLS (Figure 5b). In addition, the fact that the COS solution was slowly added to the siRNA in the ITC cell must also favor the aggregation of the complexes as discussed previously (Figure 4). For the chitosan of 90 kDa, for which no aggregation was observed at N:P < 1 (Figure 5b), the titration curve exhibited a sigmoid shape as typically found for high molar mass chitosans [3,59]. For the COS-5, the injection peaks were barely detected but the V shape was detected at the beginning of the titration, thus indicating that complexation occurred with a concomitant aggregation process (Figure 7).

The thermograms were analyzed using a modified version of the multiple non-interacting sites (MNIS) model. This model has been applied previously to the two-step complexation of a polyelectrolyte system in which small particles of the complex formed before reorganizing into a dense coacervate phase at neutrality [23]. For COS 5-13-16-50, ITC data were adjusted with the MNIS model by considering two independent contributions, the exothermic electrostatic ion pairing that can be observed throughout the titration, and superimposed by an endothermic aggregation step detected only at low N:P. Both contributions rely on a set of independent adjustable parameters, including the binding constant (K_b_), the enthalpy change per mole of injectant (ΔH) and the reaction stoichiometry (n) (see Supporting Information). The binding constant allows the determination of the free energy change, ΔG = −RT ln(K_b_) and the entropic contribution, TΔS = ΔH − ΔG. For the parent chitosan, only the exothermic ion pairing was considered since no aggregation could be detected at the beginning of the titration. The thermodynamic parameters obtained through this procedure are presented in Table 3. The ion pairing was exothermic as expected for a strongly interacting system giving a solid precipitate at neutrality in contrast to more weakly interacting systems like coacervates. These were often found to be endothermic in relation with their highly hydrated state that opposes ion pairing [38,61,65]. The enthalpy change associated with the ion pairing (ΔH) showed a marked increase (in absolute value) from COS-5 to COS-13 and then remained constant for longer COS. Similarly, the stoichiometry decreased from 3 to 1 and remained constant for higher DPs. The entropic contribution associated with the ion pairing (TΔS) was systematically higher than ΔH and regularly increased with DP. Consequently, the binding constant (K_b_) also increased with DP. The entropy change related to the ion pairing could be considered as the sum of the unfavorable entropy change caused by the fixation of the polymer chains (ΔS_p_) and the favourable entropy changes related both to the release of counter-ions (ΔS_i_ > 0) and the hydrophobic interactions (ΔS_h_ > 0) [66]. Further, ΔS_p_ should decrease with the DP because of the less significant loss of configurational entropy when the chain length increased. Whereas, ΔS_h_ which was related to the release of structured water molecules upon hydrophobic interactions of the complexed segments, should increase with the DP. As such, the hydrophobic interactions represent another level of cooperativity [67]. However, ΔS_i_ could be considered the main component of the entropy change due to the high degree of charging of COS and siRNA at pH 4.5. It can be, however, assumed that ΔS_i_ is independent of the chain length. Thus, the observed increase in total entropy change with DP was mainly due to the decrease of ΔS_p_ and the increase of ΔS_h_.

Regarding the secondary process at play, the values of enthalpy change are good indicators of the propensity of aggregation obtained during the initial stages of the titration. Accordingly, the aggregation was maximal with COS-13 and COS-16 and minimal for COS-5 and COS-50 (Table 3). The differences in the aggregation level can be explained from arguments based on the respective chain lengths and flexibilities of siRNA and COS. Considering that the siRNA is a rigid rod of 8 nm long [4], the COS-13 and COS-16 with a contour length of 6.5 nm and 8 nm and higher flexibility than siRNA as shown by their respective persistence length [68,69], can achieve an almost complete ion pairing with siRNA leading to quasi-neutral complexes without enough unpaired phosphate groups to stabilize the particles [22]. In contrast, by increasing the DP of COS to 50 and above, COS chains could bind to several siRNA chains, increasing the probability of having multiple unpaired phosphate groups that can participate in the colloidal stabilization at N:P < 1. For the COS-5, its lower affinity with siRNA must lead to incomplete ion pairing at low N:P and thus less aggregation.

## 4. Discussion

The interaction between complementary macromolecules, as oppositely charged polyelectrolytes (PEs), is a cooperative process, often explained as an entropy effect—when one of the active sites interacts with a complementary one, the neighboring sites have a more favorable entropy of binding resulting from the reduced loss of configurational entropy. Therefore, the reaction of complexation proceeds according to a zipping mechanism [66,67,70]. The stable complexes can be formed when the free energy change of the complexation exceeds the kinetic energies of the starting polymers [66]. For a cooperative process, the binding constant (K_b_) is defined as: $K_{b}^{}=K_{1}^{n}$ where $K_{1}^{}$ is the complexation constant of a monomer unit and n is the DP of the oligomer. It follows that K_b_ must depend strongly on the length of the oligomer [66,71]. Specifically, it has shown that the dependence of complex stability on the chain length of PE has a threshold character, that is, the complexation equilibrium is displaced abruptly towards the formation of the complexes when a certain critical DP is reached [66,71]. The experimental K_b_ values obtained for the ion pairing step did not completely support this prediction as K_b_ linearly increased with the DP rather than exponentially (Figure S10). This may suggest that the complexation process was not fully cooperative within the definition given above, i.e., multiple ionic pairing reactions, each characterized by its own equilibrium constant. The reason could be that the interaction between COS and siRNA was very strong, as shown by the exothermicity of the ion pairing. Therefore, the complexation reaction was likely under kinetic rather than thermodynamic control. However, the existence of a critical DP could be still evidenced in the binding isotherm where a dramatic increase in chitosan affinity for siRNA was observed when the DP increased from 5 to 13 (Figure 6). Also, the enthalpy of the ion pairing which is directly equal to the difference in Coulomb energy before and after complexation strongly increased (in absolute value) in the same range of DP (Table 3). These results agree with the work of Strand et al. where authors showed that a minimum of 6–9 protonated chitosan units were needed to provide interaction strength with DNA comparable to that of chitosan polymer [15]. It can be thus assumed that DNA and siRNA exhibit similar complexation behavior with chitosan. Interestingly, similar critical DPs have been reported for various polyelectrolyte systems: For peptides, a sequence of 6–8 cationic amino acids was required to allow strong binding with siRNA [72]; for synthetic polyelectrolytes, critical DPs between 4 and 6 were found for the system oligoethyleneimine-poly(methacrylic acid) depending on the hydrophobicity of oligoethyleneimine [73]; a critical DP of 7 was found for the complexation of poly(dimethylaminoethyl methacrylate) with oligophosphates [74].

The effect of the COS chain length on binding properties with siRNA must correlate with the solution properties of the COS alone since the variation of the pK_a_ with the DP showed the existence of a similar critical DP of approximately 10 (Figure 1 and Table 2). Therefore, the chain length influences the electrostatic properties of COS in a similar way at the intermolecular level (binding with siRNA) and intramolecular level (pK_a_). The dependence of the electrostatic properties of COS to the DP can be evaluated through the Debye screening length (λ_D_) which defines the characteristic range of electrostatic interaction in solution. In the conditions of ionic strength used, the Debye length was estimated to λ_D_ ~ 3.5 nm, which corresponds to the size of a COS segment of 7 units regularly aligned. The assumption of a rod-like conformation is compatible with the persistence length of 5 nm for chitosan [69]. Thus, the intensity of intramolecular electrostatic repulsions between neighboring charges increased between DP 1 and DP 7 resulting in a significant decrease in pK_a_ in this range of DP. Similarly, the complexation of a COS segment of 7 units with siRNA or other polyanions must considerably reduce the Coulomb energy of the system as shown by the significant decrease in the enthalpy change associated with the ion pairing step between COS 5 and COS 13 (Table 3). No significant difference in enthalpy change per mole of COS unit was detected for larger DPs, although the binding constant increased continuously with the DP, but mainly for entropy reasons as described previously. A good way to verify the dependence of the critical DP on the Debye length would be to add salt to decrease λ_D_ and see if the critical DP increases. These experiments have not been performed here. However, a similar situation can be found in the case of H-bonding complexes where the critical DP is approximately 40 as hydrogen bonds are weaker than electrostatic interactions [75]. Finally, while the requirement of having COS chains containing approximately 10 units to form stable complexes is important at the molecular level, however, this is not sufficient to achieve the colloidal stability of the complexes in solution. Specifically, it was shown that only COS of DP 50 or higher and used in excess (N:P > 5) formed small and stable colloidal complexes (Figure 5). Under such conditions, the particles are stabilized by a shell of COS chains with sufficient unpaired protonated glucosamine groups that provide electrosteric stabilization.

## 5. Conclusions

In this work, the electrostatic complexation of *N*-deacetylated chitosan oligosaccharides (COS) with siRNAs was studied under conditions of full protonation of COS and correlated with the properties of COS in solution. The behavior of COS, alone or in the presence of siRNA, is governed by the length of the chain. This has been varied here between DP5 and DP50. In particular, it was shown that, on the one hand, the binding affinity of COS for siRNA increased sharply between DP5 and DP13 and that, on the other hand, the pK_a_ of COS decreased just as abruptly between the same DP values. This highlights the existence of a critical DP from which the electrostatic interactions become particularly high, resulting in both significant repulsions between neighboring charge residues on COS chains and strong attraction with polymers of opposite charge like siRNA. Similar critical values of DP have been reported in the context of polyelectrolyte complexation which suggests that this behavior is universal. With regard to the colloidal properties of the complexes, and beyond the efficiency of the complexation, a rather high DP (~50) is necessary to provide a good stability to the complex particles through an electrosteric mechanism. These findings could apply in the context of gene delivery where both in vivo complex stability and the complex dissociation at targeted sites are needed. However, the limited solubility of *N*-deacetylated chitosan at pH 7.4, even for low DPs, remains for the moment a major difficulty in biological applications. New approaches to address this issue without altering the structure of chitosan are still relevant.

## Figures and Tables

**Table 1:** Chitosan oligosaccharides (COS) characterization by SEC-MALLS and nuclear magnetic resonance (NMR) analysis.

	SEC-MALLS					^1^H NMR
COS	*M*_n_ (g/mol)	*M*_w_ (g/mol)	Đ	dn/dc (mL/g)	DP_n_^1^	DP_n_^1^
COS-5	1030	1240	1.20	0.1660	5.4	4.5
COS-13	2290	2900	1.26	0.1752	13.2	12.2
COS-16	2710	3630	1.34	0.1860	15.8	20.2
COS-27	4560	6420	1.41	0.1863	27.3	33.0
COS-50	8190	11970	1.46	0.1801	49.8	69.7

^1^ number-average degree of polymerization of the chitosan without the M-unit.

**Table 2:** The values of pK_0_, pK_1/2_ and n of COS varying in DP (see text).

DP_n_	pK_0_	pK_1/2_	n
5	6.93	6.46	1.53
13	6.77	6.08	1.74
16	6.72	6.04	1.73
27	6.72	6.00	1.73
50	6.44	5.95	1.56

**Table 3:** Thermodynamic parameters of the siRNA/COS complexation evaluated by isothermal titration calorimetry (ITC) at T = 298 K with a multiple non-interacting sites (MNIS) model by considering two contributions, the ion pairing throughout the titration and the aggregation at initial N:P ratios. Note that the aggregation step was observed with COS 5-13-16-50 but not with the parent chitosan of 90 kDa. ΔH (kJ·mol^−1^), TΔS (kJ·mol^−1^), ΔG (kJ·mol^−1^), K_b_ (L·mol^−1^) and n denote the enthalpy change, the entropic contribution to the free energy, the free energy change, the binding constant and reaction stoichiometry (the mole refers to monomer units of COS).

	Ion Pairing					Aggregation			
	ΔH	TΔS	ΔG	K_b_	n	ΔH	TΔS	ΔG	K_b_
COS-5	−1.1	23.5	−24.5	2.0 × 10^4^	3.0	0.7	28.8	−28.1	8.5 × 10^4^
COS-13	−5.1	24.0	−29.1	1.3 × 10^5^	1.1	4.4	32.7	−28.4	9.4 × 10^4^
COS-16	−4.8	24.9	−29.7	1.6 × 10^5^	1.0	3.4	34.4	−30.9	2.6 × 10^5^
COS-50	−5.2	26.8	−31.9	4.0 × 10^5^	1.0	1.9	32.8	−30.9	2.6 × 10^5^
Parent chitosan	−4.3	29.8	−34.1	9.6 × 10^5^	1.0				

## Supplementary Materials

The supplementary materials are available online at https://www.mdpi.com/2073-4360/11/8/1236/s1.
