# Supplementary material for: Effects of Chain Length of Chitosan Oligosaccharides on Solution Properties and Complexation with siRNA
Source: Polymers (Basel). 2019 Jul 25;11(8):1236. doi: 10.3390/polym11081236 (PMC6723797; doi:10.3390/polym11081236)
Supplement: Supplementary file 1 [file polymers-11-01236-s001.pdf]

Effects of chain length of chitosan oligosaccharides on solution properties and complexation with siRNA

Supporting Information

Tim Delas [1], Maxime Mock-Joubert [2], Jimmy Faivre [2], Mirjam Hofmaier [1], Olivier Sandre [1], François Dole [3], Jean Paul Chapel [3], Agnès Crépet [2], Stéphane Trombotto [2], Thierry Delair [2], Christophe Schatz [1,*]

[1] Laboratoire de Chimie des Polymères Organiques (LCPO), Univ. Bordeaux, CNRS, Bordeaux INP, UMR 5629, 33600, Pessac, France

[2] Ingénierie des Matériaux Polymères (IMP), CNRS UMR 5223, Université Claude Bernard Lyon 1, 69622 Villeurbanne, France

[3] Centre de Recherche Paul Pascal (CRPP), UMR CNRS 5031, Univ. Bordeaux, 33600 Pessac, France

* Correspondence: schatz@enscbp.fr; Tel.: +33-5568-46618

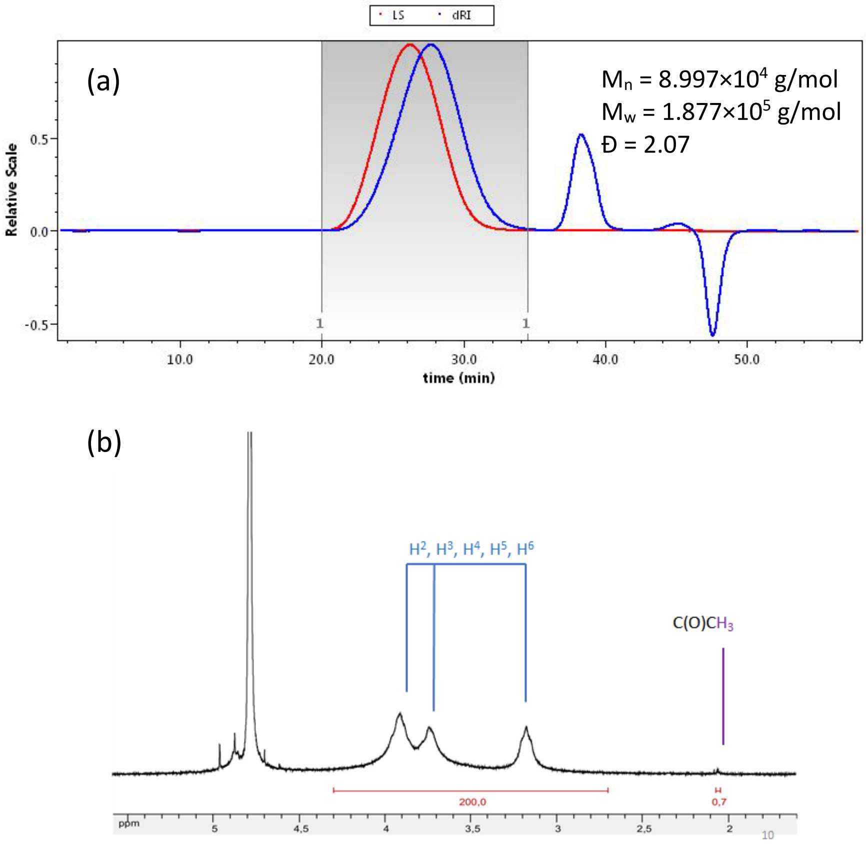

Figure S1. (a). SEC-MALLS analysis of the starting chitosan with a light scattering detection (red) and a differential refractometer (blue). (b) ^{1}H NMR analysis of the starting chitosan in D_2O.

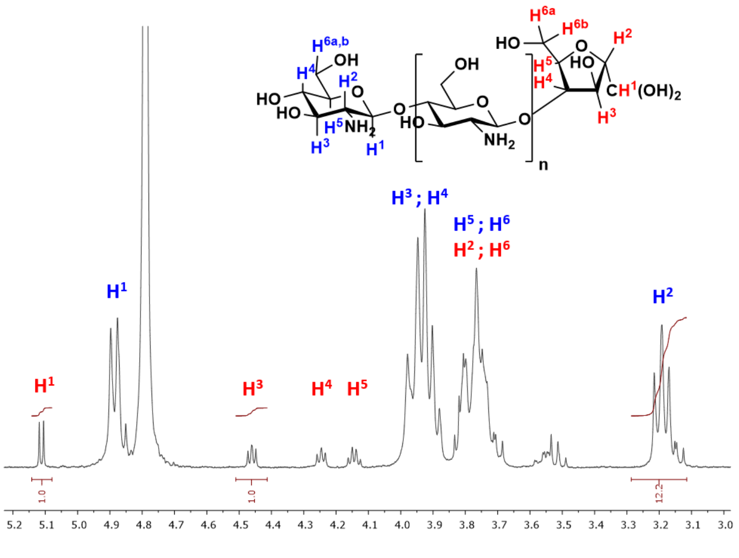

Figure S2. ^{1}H NMR analysis of the COS-13 (hydrochloride form) in D_2O.

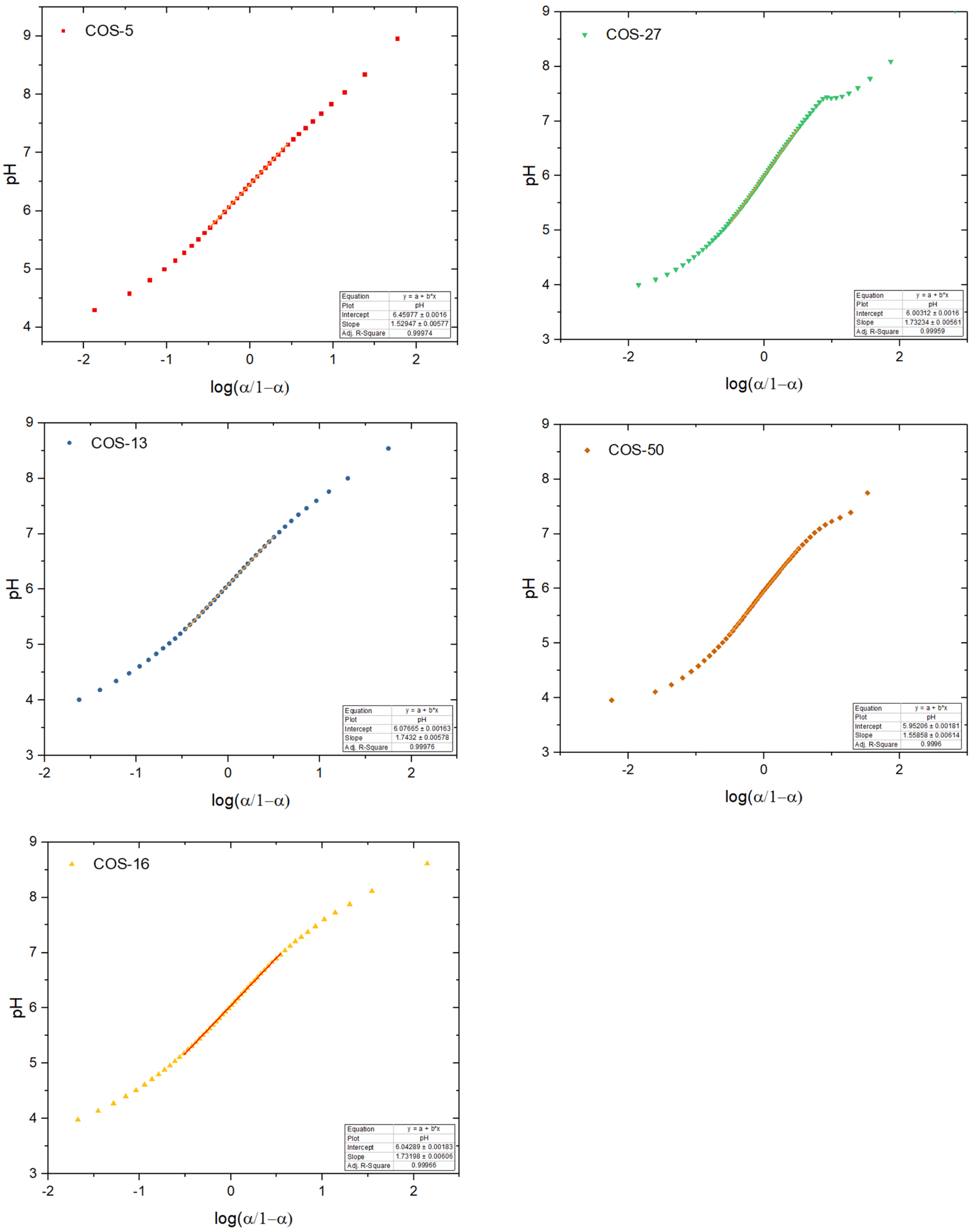

Figure S3. Determination of the $pK_{1/2}$ and n values of the extended Henderson-Hasselbalch equation, $pH = pK_{1/2} + n \log [\alpha/(1-\alpha)]$ from a linear regression in the interval $-0.5 < \log [(\alpha/(1-\alpha)] < 0.5$.

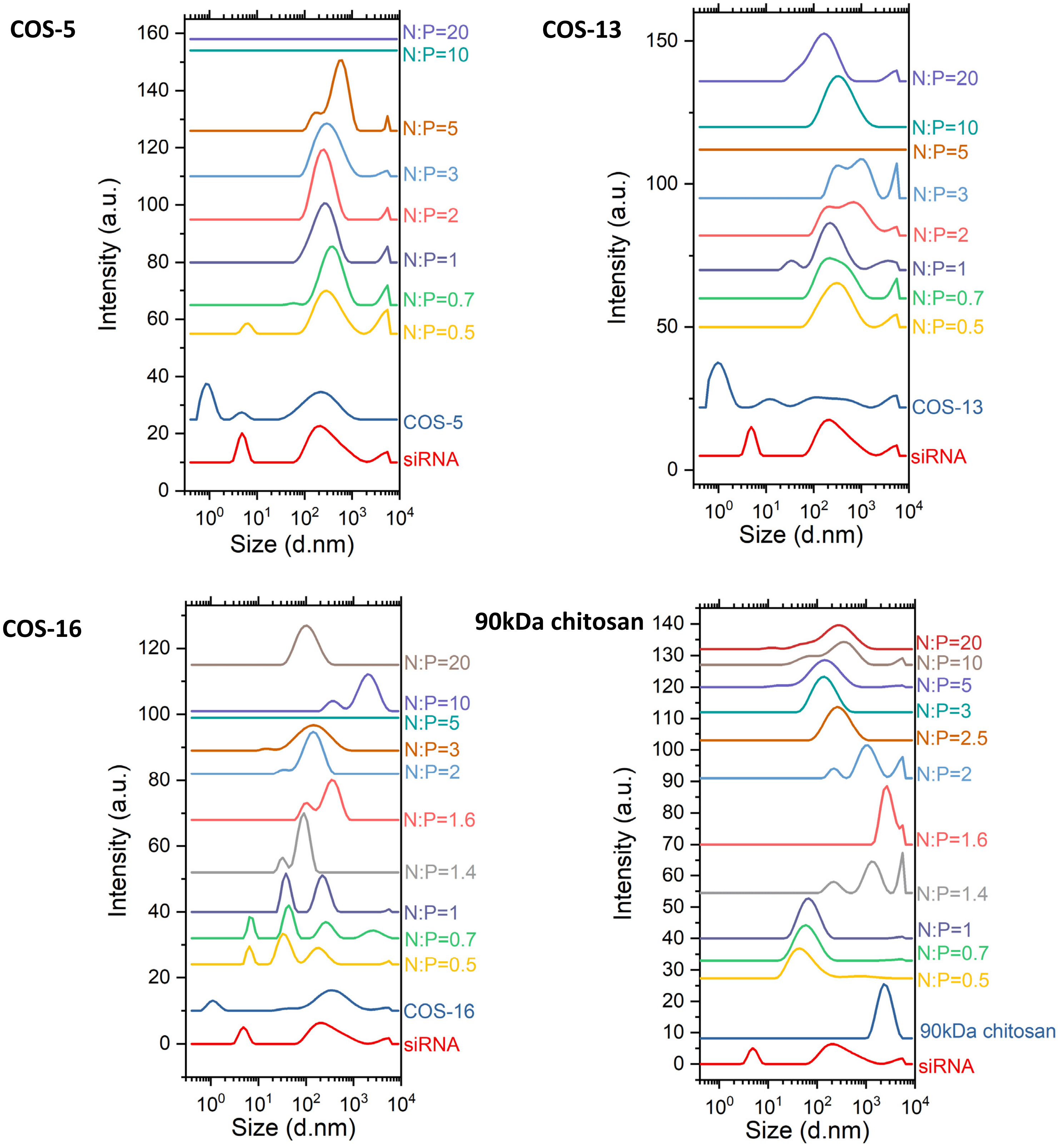

Figure S4. Intensity-average particle size distributions of COS/siRNA complexes in RNase-free water by dynamic light scattering with a 173° angle detection at various N:P ratios using a siRNA concentration of 0.1 g/L. Complexes were prepared by fast addition of the polyelectrolyte in default (COS for N:P < 1, siRNA for N:P > 1). Note that the sizes of the aggregates are out of range for COS-5 at N:P 10 & N:P 20 and for COS-13 at N:P 5.

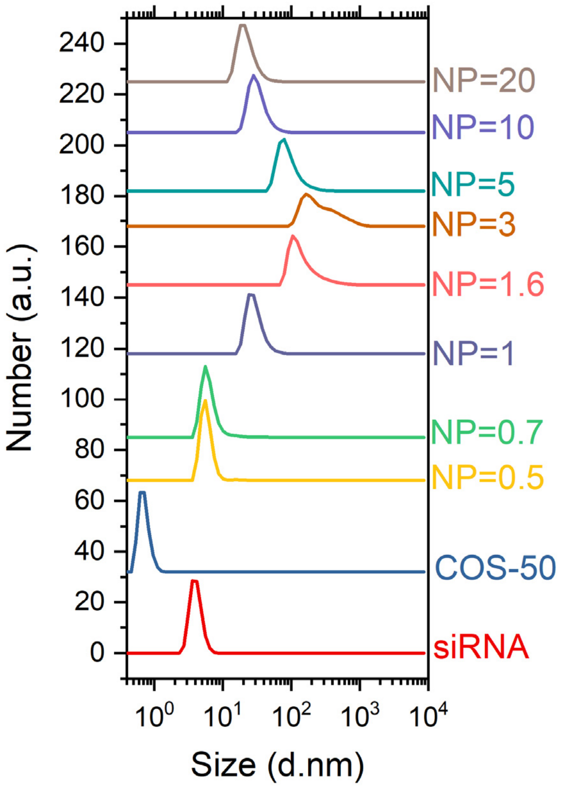

Figure S5. Number-average particle size distributions of COS-50/siRNA complexes by dynamic light scattering with a 173° angle detection at various N:P ratios using a siRNA concentration of 0.1 g/L. Complexes were prepared by fast addition of the polyelectrolyte in default (COS for N:P < 1, siRNA for N:P > 1).

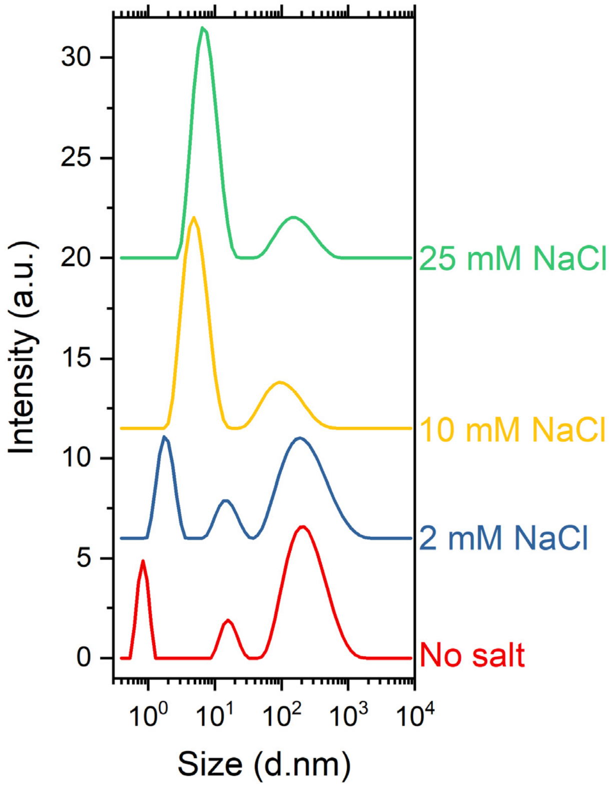

Figure S6. Intensity-average size distributions of COS-50 (hydrochloride form) in presence of various amounts of salt by dynamic light scattering with a 173° angle detection. The COS concentration is 1 g/L.

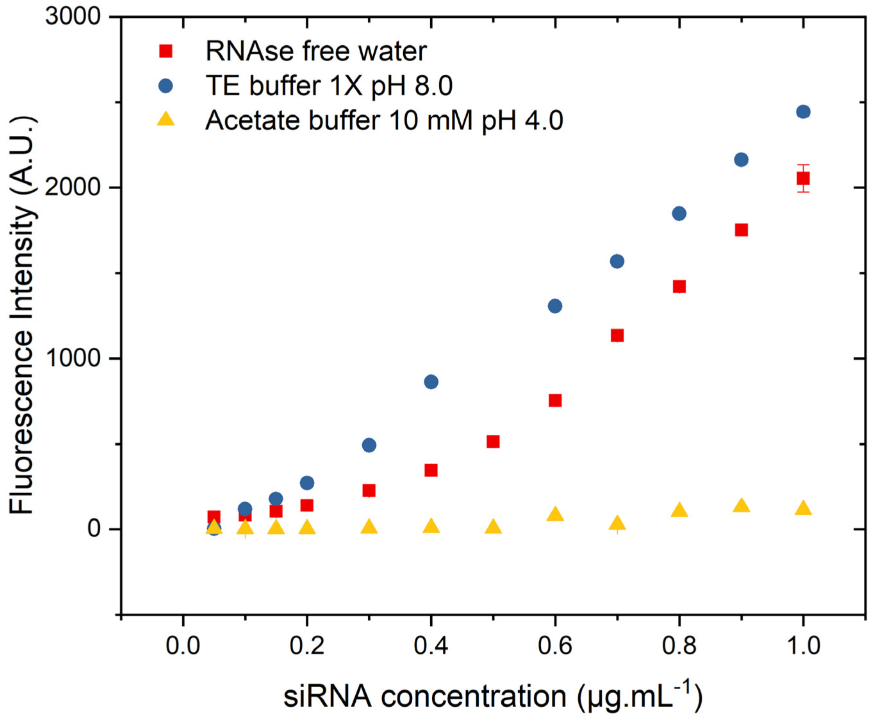

Figure S7. siRNA assay with RiboGreen in various solvent conditions (λ_{ex} = 480 nm, λ_{em} = 520 nm)

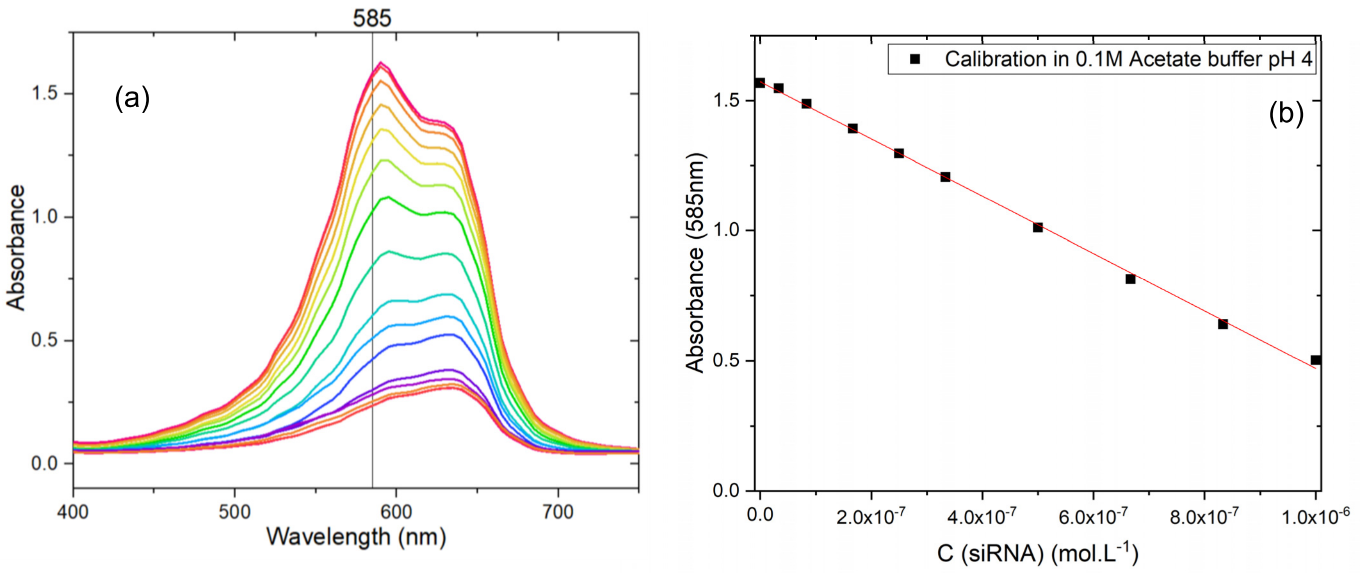

Figure S8. siRNA assay in 0.1 M acetate buffer pH 4.0 in presence of excess Toluidine Blue (TB). a) Overlay of the absorbance spectra of the supernatants after centrifugation of the siRNA/TB dispersions. b) Calibration curve of siRNA with TB at λ = 585 nm.

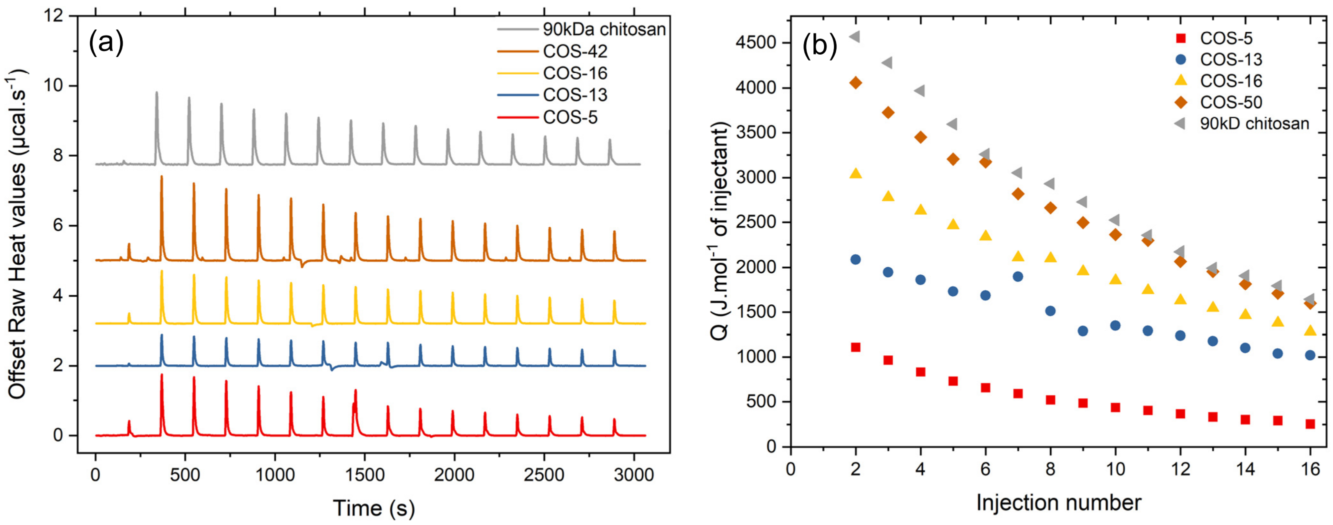

Figure S9. Heat flow per injection versus time (a) and integrated heats (b) for the isothermal dilution of COS varying in DP in 10 mM acetate buffer pH 4.5.

ITC modeling

With the exception of the parent chitosan of 90 kDa presenting a classical sigmoidal ITC isotherm, the COS of lower DP (5, 13, 16, 50) showed a more or less pronounced exothermic peak before the equivalency. A second aggregative process hindering/counterbalancing the classical ion-pairing process should then be assumed to rationalize these titration experiments. Following the approach put forward recently by Vitorazi *et al.*,[1] the heat exchange measured during the complexation of the COS with siRNA was considered to be the sum of two distinct contributions, $\Delta H_{IP}(Z, n_{IP}, r_{IP})$ for the electrostatic ion pairing process and $\Delta H_{Agg}(Z, n_{Agg}, r_{agg})$ for the aggregation process, with both contributions being of the form of equation 1 derived from the Multiple Non-Interacting Sites (MNIS) model:[2,3]

$$\Delta H(Z, n, r) = \frac{1}{2}\Delta H_b\left(1 + \frac{n - Z - r}{\sqrt{(n + Z + r)^2 - 4Zn}}\right) \qquad (1)$$

This approach supposes that the siRNA to be titrated have several anchoring sites to which COS can bind with a probability independent of the rate of occupation of the other sites on the same siRNA molecule. The complexation between siRNA and COS comes then with either an absorption or a release of heat proportional to the amount of binding. The reaction is characterized by a binding constant K_b, a binding enthalpy ΔH and a reaction stoichiometry n. In equation 1, $r = 1/K_b[M]$ with $[M]$ the molar concentration of siRNA and Z the N:P ratio. It is then assumed that the total enthalpy change during titration can be written as:

$$\Delta H(Z) = \Delta H_{IP}(Z, n_{IP}, r_{IP}) + \alpha(Z)\Delta H_{Agg}(Z, n_{Agg}, r_{Agg}) \qquad (2)$$

where the function $\alpha(Z)$ is the fraction of the aggregate phase at Z. $\alpha(Z)$ is considered to be of the form: $\alpha(Z) = \left(1 + exp((Z - Z_0)/\sigma)\right)^{-1}$ which corresponds to a step function centered at Z_0 and of lateral extension σ (with $Z_0 = n_{IP}$ and $\sigma = 0.3$ for all isotherms). The isotherm of the parent chitosan showing a classical sigmoidal variation, the binding enthalpy was fitted with a unique ion pairing process. For the chitosans of lower DPs, the two-step model was used in order to take into account the aggregation process superimposed on the ion-pairing process.

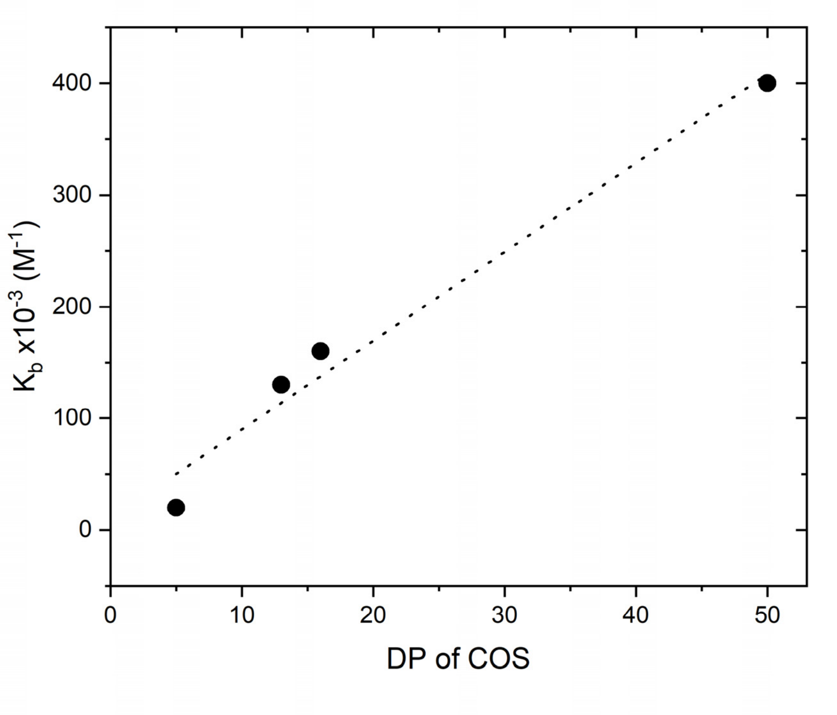

Figure S10. Binding constant related to the ion pairing as function of the DP of COS (from data in Table 3). The dotted line was plotted to guide the eye.

References

(1) Vitorazi, L.; Ould-Moussa, N.; Sekar, S.; Fresnais, J.; Loh, W.; Chapel, J. P.; Berret, J. F.: Evidence of a two-step process and pathway dependency in the thermodynamics of poly(diallyldimethylammonium chloride)/poly(sodium acrylate) complexation. *Soft Matter* 2014, *10*, 9496-505.

(2) Pierce, M. M.; Raman, C. S.; Nall, B. T.: Isothermal Titration Calorimetry of Protein–Protein Interactions. *Methods* 1999, *19*, 213-221.

(3) Wiseman, T.; Williston, S.; Brandts, J. F.; Lin, L. N.: Rapid measurement of binding constants and heats of binding using a new titration calorimeter. *Anal Biochem* 1989, *179*, 131-7.
